# Advances in Immunomodulation and Immune Engineering Approaches to Improve Healing of Extremity Wounds

**DOI:** 10.3390/ijms23084074

**Published:** 2022-04-07

**Authors:** Preeti J. Muire, Marc A. Thompson, Robert J. Christy, Shanmugasundaram Natesan

**Affiliations:** Combat Wound Care Research Department, US Army Institute of Surgical Research, JBSA Ft Sam Houston, San Antonio, TX 78234, USA; marc.a.thompson60.ctr@mail.mil (M.A.T.); robert.j.christy12.civ@mail.mil (R.J.C.)

**Keywords:** injury, wounds, trauma, inflammation, bioengineering, immunoengineering

## Abstract

Delayed healing of traumatic wounds often stems from a dysregulated immune response initiated or exacerbated by existing comorbidities, multiple tissue injury or wound contamination. Over decades, approaches towards alleviating wound inflammation have been centered on interventions capable of a collective dampening of various inflammatory factors and/or cells. However, a progressive understanding of immune physiology has rendered deeper knowledge on the dynamic interplay of secreted factors and effector cells following an acute injury. There is a wide body of literature, both in vitro and in vivo, abstracted on the immunomodulatory approaches to control inflammation. Recently, targeted modulation of the immune response via biotechnological approaches and biomaterials has gained attention as a means to restore the pro-healing phenotype and promote tissue regeneration. In order to fully realize the potential of these approaches in traumatic wounds, a critical and nuanced understanding of the relationships between immune dysregulation and healing outcomes is needed. This review provides an insight on paradigm shift towards interventional approaches to control exacerbated immune response following a traumatic injury from an agonistic to a targeted path. We address such a need by (1) providing a targeted discussion of the wound healing processes to assist in the identification of novel therapeutic targets and (2) highlighting emerging technologies and interventions that utilize an immunoengineering-based approach. In addition, we have underscored the importance of immune engineering as an emerging tool to provide precision medicine as an option to modulate acute immune response following a traumatic injury. Finally, an overview is provided on how an intervention can follow through a successful clinical application and regulatory pathway following laboratory and animal model evaluation.

## 1. Introduction

Open wound injuries are common in trauma patients. The skin relies on its natural defense mechanisms to regulate healing of minor injuries such as scrapes, cuts, insect bites and ulcers [1]. However, in traumatic extremity injuries, particularly those involving mangled skeletal musculature, healing can be severely impaired and requires highly effective treatment regimens [2]. Deep open wounds are remarkably complex as a result of several often aberrant healing cascades operating concurrently, hindering each process’ ability to function normally [3]. During the early hours post injury, several inflammatory cascades, clotting factors and immune cells are activated and directed to restore homeostasis and proper tissue/organ function [4,5,6]. In a normal healing scenario, these inflammatory cascades are regulated, allowing the ensuing regenerative cascades to proceed towards full recovery. However, in the case of delayed or impaired wound healing, the sequence of inflammatory and regenerative cascades are dysregulated, thereby skewing hemostasis and disrupting the local and systemic immune cells’ phenotype along with their functional states [5]. Such events alter the time course of the normal wound healing process, making it challenging to determine effective wound healing technologies such as effective biomaterial application and immunomodulatory molecule delivery. 

Successful wound healing requires the coordinated activities of multiple cell types that constitute the inflammatory and regenerative response following tissue injury. While mesenchymal stem cells (MSCs), endothelial cells, keratinocytes and fibroblasts have a central role in the regenerative phase, immune cells such as neutrophils, monocytes, macrophages, mast cells, T and B lymphocytes, myeloid-derived suppressor cells (MDSCs) and dendritic cells (DCs) respond to inflammatory triggers transmitted from the wound site, become activated and undergo cell proliferation, differentiation and migration [7,8]. Identifying cytokines, essential growth factors, matrix components and diverse cellular populations present at the wound site during normal and impaired healing promotes the development of promising therapeutic intervention for clinical applications. Principally, the wound healing process starts immediately after an injury, with a surge of inflammatory cells, clotting factors and cytokines, responding to external stimuli. There are many approaches to improving the healing process; however, tuning the inflammatory phase to an appropriate response, which is not found in aberrant healing scenarios, will almost certainly set the stage for optimal regeneration of the injured tissue [9]. This review outlines the immunological processes occurring during healing of cutaneous wounds following traumatic extremity injury and aims to discuss the current immunomodulatory approaches to regulate inflammation following an acute injury. Further, it enunciates the advancements in the emerging immune-targeting and immune engineering approaches applied in several wound healing and regeneration studies.

## 2. Cutaneous Wound Healing

**Figure 1:**
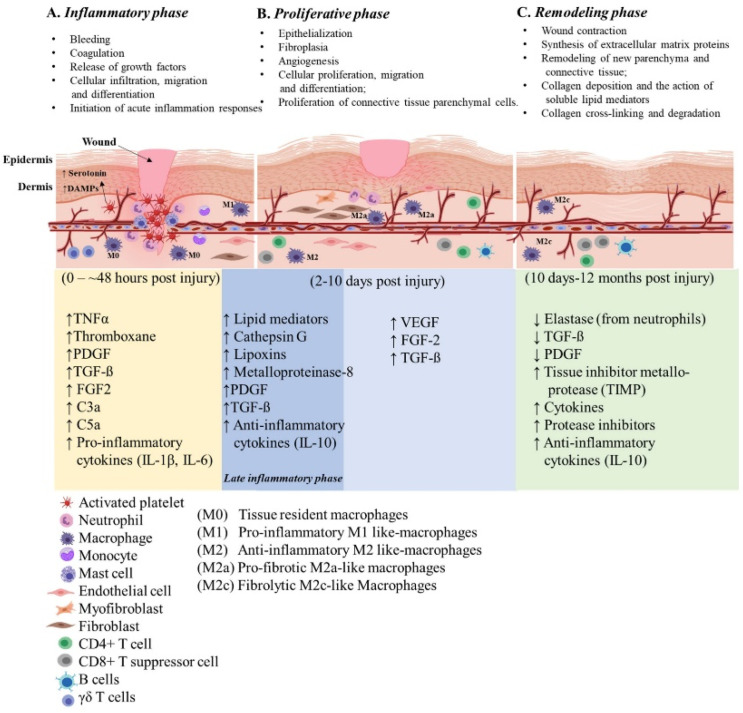
Illustration of the cutaneous wound healing process. The three phases of open wound healing are inflammatory phase: 0–~48 h (yellow area); proliferative phase: 2–10 days (blue area); and remodeling phase: 10 days–12 months (green area). Overlap of the inflammatory and proliferative phases (dark blue area) is also referred to the late inflammatory phase. The time scale starts at the time of injury and extends through 12 months post injury. The upward arrows indicate increased expression and the downward arrows indicate decreased expression of the molecules. This figure was created with BioRender.com (accessed in June 2020).

Wounds are often defined as peripheral or extensive (deeper) disruptions of the epithelial integrity of the skin, which extends to subcutaneous tissue with damage to underlying structures such as tendons, muscles, vessels, nerves and parenchymal organs [10]. Functional wound healing is a well-orchestrated sequence of overlapping phases, such as coagulation, inflammation, proliferation and remodeling. Figure 1 illustrates the sequence of wound healing events following an acute injury.

### 2.1. Inflammatory Phase

Soon after the injury, platelets and neutrophils infiltrate the injury site and create contact with exposed collagen and extracellular matrix (ECM) components to form a fibrin clot. This contact triggers platelets to release tumor necrosis factor α (TNFα) (a pro-inflammatory cytokine), thromboxane (platelet-activating factor), clotting factors, as well as platelet-derived growth factor (PDGF) and transforming growth factor-beta (TGF-β) (essential growth factors) to initiate healing cascades that mark the beginning of hemostasis. Platelets also release vasoactive amines, such as serotonin, which signals vasodilation and increases vascular permeability, leading to fluid extravasation and edema. As the blood components spill into the injury site, platelets release vasodilators and chemo-attractants and initiate the release of complement anaphylatoxins, most importantly C3a and C5a [11]. While platelets attract neutrophils and contribute to the local inflammatory milieu from the initial insult, they are also responsible for laying the groundwork for healing. Platelets act as promoters in the wound healing cascade to attract and activate macrophages, endothelial cells and fibroblasts [12]. The platelet derived factors PDGF and TGF-ß promote chemotaxis of neutrophils, fibroblasts and smooth muscle cells [13], recruits and activates naïve monocytes and macrophages and maintains an immunological balance through the inhibition of differentiation and survival of lymphocytes [14].

Mast cells, a myeloid granulocyte, have a pivotal role in the regulation of wound healing and fibrosis [15]. Increased densities of mast cells are observed at the wound site and they interact with and activate the present fibroblasts [16]. Mast cells release granules filled with enzymes, histamine and other active amines; these mediators are responsible for the characteristic signs of inflammation around the wound site [17]. The active amines released from mast cells induce endothelial permeability, resulting in the extravasation of mononuclear cells and edema at the site of injury, contributing to the development of characteristic signs of inflammation. Neutrophils release chemical signals such as cytokines and growth factors needed to recruit other immune and connective tissue cells to promote neovascularization [18]. Neutrophils also release significant amounts of soluble factors such as cathepsin G, lipoxins and enzymes such as collagenase (matrix metalloproteinase-8/MMPs) responsible for the remodeling of the provisional connective tissue matrix during the healing process [19]. Cellular release of specialized pro-resolving modifiers such as lipoxins derived from arachidonic acid and the production of resolvins, mareisins and protectins from omega-3 fatty acids help drive resolution of inflammation and clearance of cellular debris [20,21]. Typically, neutrophils will engorge themselves with foreign bacteria and host cell debris at the wound site until they are filled and constitute “laudable pus” in the wound [22,23]. The neutrophil extracellular traps (NETs) aid in clearing damage-associated molecular patterns (DAMPs) as well as necrotic cellular debris [24]. During uneventful wound healing, neutrophils are cleared from the wound area by β2 integrin signaling and macrophage phagocytosis [25,26].

Approximately 48 h post-injury (hpi), resident monocytes receive signals to polarize towards a wound macrophage phenotype. Wound macrophages display a unique phenotype that partly retains functional aspects of the originator monocytes, sharing characteristics of pro- and anti-inflammatory macrophages [27]. These specialized wound macrophages are perhaps the essential early pro-inflammatory cells involved in the normal healing response [28]. Once activated, these wound macrophages release additional PDGF and TGF-ß to promote migration of fibroblasts and smooth muscle cells to the wound site [29]. Neutrophils promote circulatory monocyte infiltration [30] by upregulation of monocyte chemoattractant protein-1 (MCP-1) and help to reprogram M1 macrophages to their M2 phenotype [31]. M1 macrophages are responsible for phagocytosing necrotic and apoptotic cells (a concept known as efferocytosis) [32], bacteria-filled neutrophils, damaged matrix, foreign debris and any remaining bacteria from the wound site. Ideally, the presence of M1 macrophages indicates that the inflammatory phase is nearing its end and the proliferative phase is beginning.

Macrophages, platelets, keratinocytes, lymphocytes and fibroblasts release additional PDGF, fibroblast growth factor (FGF), TNFα and IL-1 (interleukin-1) induced by TGF-β signals. TGFβ, FGF and TNFα released by platelets and inflammatory cells play an essential role in both the initiation of inflammation and regulation of fibroblast activating signals [33]. TGFβ released from macrophages stimulates differentiation of myofibroblasts and increases the synthesis of matrix proteins such as collagen, proteoglycan and fibronectin. During the later phase of wound healing, neutrophils assist in recruiting T cells by upregulation of chemokine ligand 3 (CCL3) [34]. Additionally, circulating T and B lymphocytes infiltrate into the wound site during the late inflammatory phase and remain there throughout the healing process in moderate to low numbers [35].

### 2.2. Proliferative Phase

Once the wound site is debrided, the proliferative phase begins and lasts for approximately 5 days, depending on the depth of the wound. During this phase, fibroblasts, which are cells of mesenchymal stem cell (MSC) origin, migrate towards the wound area, deposit new ECM [36,37] and restore functionality of the injured tissue. Appropriate fibroblast activity is crucial to initiate tissue injury repair while the collagen laid down by fibroblasts provides structural integrity as it later becomes crosslinked and organized during scar formation [38]. Within a few days, fibrin fills in the defect which is characteristic of a scab formation. At this time epithelial regeneration occurs beneath the scab and is accompanied by the formation of granulation tissue and angiogenesis (new blood supply).

During the late proliferation phase, several other biological processes that drive epithelization become activated. The epithelization process is stimulated by the presence of epidermal growth factor (EGF) and TGFα, which are produced by activated wound macrophages, platelets and keratinocytes [13]. Upon completion of the initial epithelial bridge, systemic proteolytic enzymes dissolve the attachment at the base of the scab resulting in its removal. During this phase, the wound site is deprived of oxygen (hypoxia) and nutrients mainly due to the high enzymatic-metabolic activity of the healing tissue. The wound microenvironment potentiates angiogenesis, mediated by factors such as low pH, reduced oxygen tension and increased lactate [39]. Additionally, angiogenesis or neovascularization, is also stimulated by vascular endothelial cell growth factor (VEGF) [40], FGF [41] and TGFß [42], all secreted by epidermal cells, fibroblasts, macrophages and vascular endothelial cells. Another important factor released by the vascular endothelial cells under the influence of hypoxia is hypoxia-inducible factor (HIF), a nuclear transcription factor [43], which regulates the expression of VEGF and stimulates angiogenesis. As new blood vessels enter the wound repair area, normoxia is achieved, and oxygen binds to HIF and blocks its activity leading to a decreased synthesis of VEGF, creating a feedback mechanism.

### 2.3. Remodeling Phase

This phase includes wound contraction and collagen crosslinking, making up the final steps in wound healing, i.e., remodeling. This process is orchestrated by cytokines and growth factors released by local and infiltrating cells. TGFβ plays a vital role in the early stages of remodeling. It inhibits collagen degradation and promotes wound contracture by inducing the expression of tissue inhibitor metalloprotease (TIMP) [44] and other protease inhibitors, limiting the enzymatic activity of proteases that target collagen. For a successful remodeling phase, secretion of important healing factors such as PDGF and TGFβ must be dampened so that the activity of these proteases is restored. To accomplish this, elastase, an enzyme secreted by neutrophils, cleaves PDGF, TGF-β and other cytokines [45]. The terminal stage of collagen remodeling is usually accompanied by collagen degradation [46]. Collagenases produced by fibroblasts, neutrophils and macrophages are responsible for the enzymatic degradation of the collagen matrix. Gradual decline in the overall T cells and increase in CD8^+^ suppressor T cells and B cells occur during later stages of wound healing. The rise in CD8^+^ suppressor T cells has a role in stopping the healing process, which is vital during wound closure [35].

## 3. Delayed Cutaneous Wound Healing

In the case of severe traumatic injuries, the stringent processes of inflammation and resolution can become dysregulated. A delay in the macrophage polarization steps (i.e., M0 → M1 → M2) post-injury via therapeutics, stress, disease or trauma will also delay the healing response [47]. One of the hallmarks of chronic wounds is the upregulation of anti-apoptotic genes [34] which increases circulating half-life and rolling speed of neutrophils (transform from physiologic phenotype to pathologic phenotype) to the injury site and thus releasing proteolytic enzymes such as neutrophil elastase, whose function is to destroy growth factors and anti-inflammatory cytokines in the milieu [45]. Additionally, in their pathologic phase, overactive neutrophils undergo high NETosis and display a dysfunctional phenotype characterized by increased infiltration, altered cytokine profile and increased reactive oxygen species (ROS) production, as well as impaired antimicrobial defense and phagocytic activity. This persistent presence of neutrophils is one of the leading factors of delayed resolution of inflammation [48]. The perpetuated inflammatory state increases leukocyte recruitment to the wound; their presence leads to a greater local release of ROS [49]. ROS degrade growth factors involved in healing and decrease the availability of these molecules [50]. The overproduction of ROS causes cellular damage via the oxidative modification of most biological macromolecules limiting or eliminating their biological activity [51]. More specifically this interference affects the differentiation and proliferation of fibroblasts and keratinocytes, cells essential for wound remodeling, often resulting in apoptosis [52,53]. Similarly, the out-of-control infiltration of neutrophils results in the lack of appropriate signaling cues for cell–cell interactions and delays the healing process until the persistent chronic inflammation is regulated. Like the myeloid populations, the lymphoid populations are also modulated during wound healing [54]. Additionally, the persistent release of DAMPs from necrotic tissue and cytokine synthesis in the wound site can dysregulate the inflammatory response, thereby causing damage to the surrounding healthy ECM inhibiting efficient wound healing [55].

Pro-inflammatory cytokines increase matrix metalloproteinase (MMP) and other protease production, impairing cell proliferation and migration, and consequently decreasing the accumulation of ECM components [56,57,58]. Tissue inhibitors of metalloproteinases (TIMPs) are also reduced [59] contributing to protease deregulation in cutaneous injuries [60,61]. This results in degradation of the important ECM components collagen, elastin and fibronectin, among others. In addition, the imbalance of pro- and anti-angiogenic factors also inhibits neovascularization and blood flow in the area [62], delaying the subsequent mechanisms for proliferative and remodeling phases. Fibrosis and chronic non-healing wounds are grouped under impaired wound healing. Collagen provides strength, integrity and structure and is needed to repair the wound defect and restore anatomic structures and functions. Deleterious deposition of collagen in the wound site leads to fibrosis, reduced remodeling and tissue architecture and loss of function. Fibrosis is typically characterized by excessive matrix deposition and fibrotic lesions are often associated with increased densities of mast cells [16]. Conversely, if an insufficient amount of collagen is deposited, the structural integrity of the wound is compromised and may lead to dehiscence. Collagen deposition and degradation are controlled by the secretion of growth factors such as PDGF and TGFβ from platelets, inflammatory cells and other vascular epithelial cells (Table 1). A lack of re-epithelialization is an ensuing outcome of delayed or non-healing wounds. As a consequence of chronic inflammation and reduced vascularization, keratinocytes from wound edges acquire a hyper-proliferative state due to the overexpression of the ß-catenin and c-myc pathway [63], ultimately leading to scarring. Poor migratory potential of keratinocytes is also related to the proteolytic degradation of growth factors and ECM proteins that are required for migration [64].

## 4. Deep Soft Tissue Injuries

Traumatic extremity soft tissue injury may occasionally extend beyond skin involving underlying muscle layer. In such an event, regeneration of skeletal muscle after traumatic injury begins with partitioning healthy from damaged and unrecoverable tissue. Damaged muscle fibers with an intact basal lamina are often salvageable; however, severe crushing or lacerations resulting in mangled tissue may disrupt the basal lamina enough to induce necrosis [77]. Subsequent activation of the complement pathway (C3a and C5a) has been shown to lead to muscle cell lysis as well as activation of chemotactic factors and leukocyte infiltration [78]. Extravasation into the wound site by these cells leads to the critical step of phagocytosing cellular debris. Ideally, once the wound is effectively cleared of necrotic wound debris, revascularization becomes key as insufficient muscle regeneration is often linked to incomplete or insufficient tissue vascularization. For example, after wound debridement and graft transplantation for significant muscle trauma it has been found that the central region of the graft is most likely to fail or become fibrous due to neurovascular connections being unable to penetrate the depth of the tissue [79]. Neovascularization is heavily supported by FGF and VEGF, among other pro-regenerative factors [80]. After creating the vascular groundwork to support nutrient and waste transport, activation of satellite cells and muscle precursor cells further the regeneration phase. Muscle precursor cells and fibroblasts support the synthesis of ECM components required for muscle innervation and function, including laminin, collagen and fibronectin [81]. The formation of these key ECM structural components leads to improved cell motility and proliferation, supporting the re-growth of healthy skeletal muscle. 

In aberrant cases of healing, neutrophils and other inflammatory cells recruited in excess produce greater levels of MMPs such as MMP-9 which, as previously discussed, inhibits ECM formation that is critical to myofiber organization and function [82,83]. The recruited neutrophils also release chemoattractant cytokines, which promote further infiltration of monocytes and macrophages. Metabolic imbalances and a pro-inflammatory state disrupt the balance of M1 and M2 macrophages, subsequently increasing the expression of fibrosis-promoting growth factors such as epidermal growth factor (EGF), VEGF and TGFβ [84,85,86]. TGFβ activates resident fibroblasts and inhibits fibro-adipogenic progenitors (FAPs) apoptosis, as well as induces their later differentiation to a fibrogenic lineage, leading to excessive ECM deposition and fibrosis. In addition, PDGF receptor-beta positive (PDGFRβ^+^) cells, which are mesenchymal pro-fibrotic cells, proliferate and differentiate to myofibroblasts post-injury via the activation of αv integrins [87,88]. Fibrosis deteriorates both structural and functional properties of skeletal muscle and affects muscle fiber regeneration after injury. In addition, fibrosis increases muscle susceptibility to re-injury.

Apart from myofiber formation, restored skeletal muscle function is dependent on sufficient vascular and axonal recovery. After injury, activated endothelial cells detach from their neighboring cells, through disruption of vascular endothelial cadherin junctions [89], resulting in increased vascular permeability. The endothelial basement membrane is degraded by proteolytic enzymes such as MMPs, releasing matrix-bound angiogenic factors that, in turn, stimulate endothelial cell migration and proliferation [90]. Capillary tube formation, deposition of a new basement membrane and anastomosis lead to blood flow. An interesting balance between angiogenesis and inflammation has been noted in some cases. Increased permeability of newly formed [91,92] leading to a prolonged inflammatory state [93] and delayed skeletal muscle healing [94]. 

Regardless of the extent of soft tissue injury, inflammation plays a pivotal role in directing the healing progress. Therefore, resolving inflammation impels considerable focus to allow positive healing and regeneration of an extremity trauma injury. Further, this review continues to delineate the established immunotherapies that are currently used; under investigation and the potential advanced treatment modalities for future consideration. Most of the existing and currently investigated immunotherapies are applied to treat a group of targets within the inflammatory milieu and we categorize them as ‘immunomodulatory therapies’. The newer generation of therapeutic approaches aim at regulating immune response through specific targets or a family of molecules, and hence are tailored to address specific inflammatory conditions at a precise magnitude, time and spatial domain. Therefore, the newer generation treatment approaches are categorized as ‘immune engineered therapies’.

## 5. Current Immunomodulatory Approaches 

**Figure 2:**
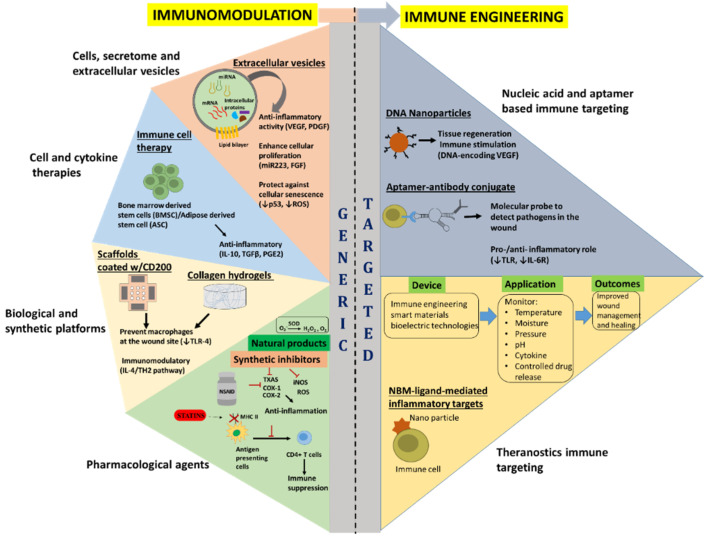
Overview of the current and emerging immunomodulatory (generic) and immune engineering (targeted) approaches for wound healing. Vascular endothelial growth factor (VEGF); Platelet derived growth factor (PDGF); Fibroblast growth factor (FGF); Interleukin (IL)-10; Transforming growth factor β (TGFβ); PGE2; Toll-like receptor 4 (TLR-4); Thromboxane A2 synthase (TXAS); Cyclooxygenases 1 and 2 (COX-1 and COX-2); Inducible nitric oxide synthase (iNOS); Reactive oxygen species (ROS); and Superoxide dismutase (SOD). The red T shaped symbols indicate inhibition or blocking of that particular molecule/process.

Regulation of the immune response to a desired level through modulation of immune response determinant factors has been widely investigated through pharmacological agents such as glucocorticoids or related agents, non-steroidal drugs, antibodies, cytokines, immunoglobulins and cellular therapies (Figure 2) [95,96]. A summary of select immunomodulatory approaches and their applications can be found in Table 2. 

### 5.1. Pharmacological Agents

Synthetic or semisynthetic drug-based therapies to combat inflammation post-trauma are often the most practical when considering the logistics of their application. The time and resource expenditure of cellular-based therapies and protein-based therapies, for example, are significantly greater compared to that of pharmacological agents with regards to synthesis, isolation, storage considerations and cost. The current standard for clinical care is the use of non-steroidal anti-inflammatory drugs (NSAIDs). NSAIDs possess anti-inflammatory, antipyretic, analgesic and thrombotic properties due to their inhibition of cyclooxygenases 1 and 2 (COX-1 and COX-2) [112,113]. From an inflammatory standpoint, COX-1 and -2 initiate the formation of prostaglandins which induce the cardinal signals for acute inflammation [114]. Successful use of NSAIDs after traumatic injury was found in a prospective review of clinical data collected from 73 adult combat casualties, several of which received NSAIDs post-operatively after surgical wound debridement [97]. The full range of NSAIDs used was not provided; however, the results, supported by use of similar demographics and wound characteristics, suggested that patients receiving NSAIDs had significantly decreased concentrations of inflammatory cytokines IL-2, IL-6, IL-8 and monocyte chemoattractant protein-1 (MCP-1) [97]. On a cautionary note, aspirin and non-aspirin NSAID use and subsequent inhibition of cyclooxygenase blocks the formation of thromboxane A2; the lack of thromboxane-dependent platelet aggregation may consequently prolong bleeding time. Therefore, extremity trauma involving hemorrhage may result in deleterious effects stemming from NSAID use, if incorporated too early. In addition, specific forms of non-aspirin NSAIDs used at high doses or over prolonged periods have also displayed a higher incidence of myocardial infarction and stroke [115,116]. 

General cyclooxygenase inhibitors are also prevalent within the clinic. COX-2 inhibitors are more common than COX-1 inhibitors as they have been found to adversely affect the gastrointestinal mucosa [117]. COX inhibitors are routinely used post-operatively to transiently relieve inflammation [118]. Recent studies have supported the use of COX-2 inhibitors not just for their analgesic properties; however, some studies have shown accelerated limb functional recovery, in vivo and post-injury [98]. In the same vein as COX-1 and COX-2, thromboxane A2 synthase (TXAS) produces arachidonic acid metabolites, specifically thromboxane A2 (TXA2), with implications in inflammation. TXA2, via its specific thromboxane prostanoid (TP) receptor, stimulates platelet aggregation and vasoconstriction after injury, often resulting in portal hypertension and further damage [119]. TXA2 activates TP to enhance protein kinase C (PKC) which, downstream, negatively affects nitric oxide production as well as ROS production [120]. Targeting TXAS with inhibitors such as prostaglandin H2 or Dazmegrel (UK-38,485), combined with TP receptor antagonists have shown a greater anti-platelet and therefore a greater anti-inflammatory effect than even some low-dose aspirin therapies [121]. 

As it pertains to deep tissue trauma, recent studies have delved into countering the effects of pro-inflammatory oxidizing radicals; ROS and reactive nitrogen species (RNS) being the two prime examples specifically involved in inflammation stemming from cutaneous and skeletal muscle injury. Inducible nitric oxide synthase (iNOS), for example, increases acetylation and activation of p65, p53 and nuclear factor kappa-light-chain-enhancer of activated B-cells (NF-κB) [122], central mediators of pro-inflammatory gene induction and function for both innate and adaptive immune cells. Downstream, burn injuries induce robust expression of iNOS in skeletal muscle, subsequently increasing apoptosis in myofibers [123]. As a result, studies have attempted to inhibit the pro-inflammatory effects of these radicals. Currently, iNOS inhibiting agents such as N-[3-(aminomethyl) benzyl] acetamidine (1400 W) and heat shock protein 70 (HSP70) have successfully reduced the impact of iNOS through different suggested mechanisms [99,124,125], showing improvements in cutaneous and muscle injury outcomes [124,126]. A study using a rodent model of volumetric muscle loss demonstrated that administration of retinoic acid receptor-γ agonist (RAR-γ), an immunomodulator, adequately improved the neuromuscular function of the injured muscle, suggesting the therapeutic potential of RAR-γ for effective muscular skeletal regeneration [127]. 

Superoxide therapies are also gaining traction as a potential therapeutic to directly ameliorate the pro-inflammatory effects of ROS while supporting antioxidant effects. Superoxide dismutase (SOD) specifically catalyzes the conversion of the superoxide anion free radical (O_2_−) to hydrogen peroxide (H_2_O_2_) and molecular oxygen O_2_; H_2_O_2_ is subsequently reduced to water by the catalase enzyme [128]. H_2_O_2_ is an essential indicator of redox metabolism, with its elevation and decrease being directly associated with the inflammatory response [129] post-injury. In conjunction with mechanical and physical modalities of wound healing [100], manganese superoxide dismutase therapy has been shown to support antioxidant capacity, while reducing pro-inflammatory angiopoietin-2 and increasing pro-regenerative VEGF in a rodent model of traumatic full-thickness cutaneous injury [100]. Statins can also inhibit inflammation through their demonstrated ability to reduce the production of inflammatory markers such as the C-reactive protein or serum amyloid A [130]. In addition, statins may decrease antigen presentation and T cell activation by restricting expression of the major histocompatibility complex class II (MHC-II) as well as reducing the cell-surface expression of other immunoregulatory molecules such as CD4, CD8, CD28, CD40, CD80 and CD54 [131]. Currently in vitro and in vivo studies support statins’ ability to impair natural killer cells and T lymphocyte proliferation and cytotoxicity, possibly through the inhibition of cellular adhesion molecules on leukocytes and endothelial cells, thus limiting migration to the wound site. Although effective, there is evidence that rare cases lead statins to induce myotoxicity and/or autoimmune myopathy [132]. To date, several statins have been investigated with some, such as Food and Drug Administration (FDA)-approved Atorvastatin, resulting in improved wound healing as well as post-injury graft integration graft integration post-injury [133].

The use of topical insulin to manage wound inflammation is a practice dating back to the 1970s [134]. It has been shown that the insulin receptor, IRS-1, IRS-2, ERK and Akt pathways are all impacted by insulin and significantly dysregulated upon injury [135,136], indicating a potential role of the insulin signaling pathway in wound repair. Insulin has previously shown anti-inflammatory properties by increasing IL-4, IL-13 and Il-10 production and decreasing IFN-γ production in injuries [135], with no adverse events or indications of hypoglycemia. Supporting the healing mechanisms of insulin, Rezvani et al. [137], performed a randomized, double-blind, placebo-controlled trial to determine the effect of topical insulin in 45 patients with non-infected acute and chronic extremity wounds. Subjects were randomly administered crystalline insulin sprays (10 U) or saline solution twice daily. A significant increase in wound healing rate (46.09 mm^2^/day vs. 32.24 mm^2^/day) was found in insulin treated patients independent of baseline wound size, again with no signs or symptoms of hypoglycemia. 

Naturally derived products are also currently being investigated with some success. In aloe vera, the mucilaginous gel present in the leaves of the plant with the same moniker, has been used from for centuries for its anti-inflammatory properties. The mechanism of action for promotion in wound healing is based on inhibition of ROS production, prostaglandins and cytokines [138]. Honey, similarly, has an extensive history of use. The anti-inflammatory action of the honey is due to inhibition of several factors including inhibiting ROS formation, complement pathway, leukocyte infiltration, COX- 2, iNOS and matrix metalloproteinase-9 (MMP-9) [139]. More recently curcumin, a product of plants from the Curcuma longa species and a derivative of turmeric, has gained more traction. Its mechanism of action has been associated with decreased expression of pro-inflammatory TNF-α, IL-1β and MMP-9, and increased levels of the anti-inflammatory cytokine IL-10, as well as providing antioxidant enzymes to the wound site [140]. It should be noted that these natural therapies are more closely associated with topical applications and have not garnered widespread clinical use to this point. 

### 5.2. Biological and Synthetic Platforms

Scaffold-based strategies to improve wound healing have proven not only beneficial, but possibly essential for severe traumatic injuries involving a significant loss of tissue, enervation and vascularization. Prior clinical practices of delivering cell suspensions directly into a wound defect site proved largely ineffective due to insufficient cellular retention within the wound and payload flushing into the surrounding tissue [141]. Consequently, the implementation of durable materials with advantageous mechanical, chemical and biologically relevant properties, are an attractive option to circumvent the limitations of native healing and direct cell-based therapies [142]. From this initial scaffold foundation, bioactive additives such as proteins, carbohydrates, small molecules or peptides are incorporated to further stimulate cell function and tissue development.

With the benefits provided by scaffolds come commensurate biocompatibility considerations to ensure implants elicit no immune response of their own. Unexpected variables in the implant material or its design can have significant negative impacts with relation to the immune response [143,144]. Historically, the biocompatibility of these implants centers around avoiding foreign body response (FBR) and fibrotic encapsulation, pro-inflammatory cues stemming from mechanical or chemical incompatibilities and toxicity of degradation products. Similar to most inflammatory responses, neutrophil recruitment is a first step in FBR, as they attach themselves to the provisional matrix. Infiltration of neutrophils is followed by the influx of monocytes and macrophages, with subsequent remodeling via collagen deposition and capillary bed formation, mediated by fibroblasts [145,146]. Aberrant FBR is classically characterized by the arrival and fusion of macrophages around the foreign body to form giant cells [147]. Introduction of foreign objects can amplify inflammation as a result of the accumulated neutrophils and macrophages at the injury site. The then markedly pro-inflammatory microenvironment consequently limits the integration of biomaterials with surrounding native tissue.

The material basis of scaffolds is just as important as any bioactive payload as it will likely comprise the largest and most sustainable portion of any implanted scaffold. The focus of implantable biomaterials in its earliest stages began with bio-inert implants, but has since shifted to bioactive designs, highlighting complex interactions between cell physiologic systems and material properties. Since then, many novel strategies have been developed to prevent the fibrous capsule formation by changing the polarity, hydrophobicity, topography and surface chemistry of the implanted materials [147], with many studies finding that, with regards to soft tissue injuries, biological scaffolds produce significantly improved results. It has been noted that biological scaffolds do not induce an FBR response as synthetic materials do [148,149]. 

Despite the early response of neutrophils to scaffold implantation and known inflammatory impact, recent evidence suggests that neutrophil activation does not directly alter the fibrotic response to biomaterials [150], rather, neutrophils set the stage for a pro-fibrotic response by macrophages by secreting a milieu of inflammatory cytokines. However, depletion of neutrophils has proven to limit healing despite their pro-inflammatory complications [151]. There is further evidence that early immune responses by neutrophils are important for the downstream modulation of T helper type 2 cells (Th2) and M2 macrophages relevant to complete wound healing [152,153]. As an alternative, therapies have aimed to limit neutrophil recruitment or activation. It has been shown previously that pre-coating a modified CD47 on polyvinyl chloride surfaces was able to reduce recruitment and adhesion to the biomaterial surface [154]. Abaricia et al. demonstrated that altering material surface characteristics and topography also plays a critical role in modulating neutrophil activity. Pro-inflammatory cytokine release as well as the production of NETs was decreased upon in vitro culture of neutrophils on rough hydrophilic titanium surfaces. Conditioned media from the neutrophils was used to culture macrophages and also produced a less pronounced pro-inflammatory state compared to macrophages treated with media from neutrophils cultured on smooth hydrophobic titanium surfaces [102]. Results of these studies are also conflicting, however, as neutrophil adherence to roughened materials has also been reported to trigger a more rapid production of ROS compared to smooth materials, suggesting that the inflammatory response can also be exacerbated [155]. 

Macrophages are considered essential to modulating an appropriate response to scaffold implantation due to their plasticity, and can subsequently change their phenotype in direct response to cytokine milieu present in the microenvironment. Inability to resolve chronic inflammation triggers fusion of macrophages to form giant cells, fibroblast recruitment, excessive collagen deposition and resolution through fibrous capsule formation. Using a streptavidin-conjugation technique, Kim et al. showed that materials coated with the endogenously expressed immunomodulatory molecule, CD200, effectively suppressed the influx of macrophages, thus dampening the inflammatory response to foreign materials in both in vitro and in vivo models [156]. It was later determined that the suppression occurred via Toll-like receptor 4 (TLR-4) downregulation [157]. Heparin is another biomaterial coating that limits the ability to form a fibrous layer by binding anti-thrombin, thus inhibiting the coagulation cascade [103]. IL-4 has been implicated in directing tissue repair, regeneration, as well as fibrosis, providing conflicting benefits and detriments to reducing inflammatory responses. It was shown that Th2 cells play a critical role in skeletal muscle injury by promoting the M2 macrophage phenotype; mediated specifically by IL-4. IL-4 has been implicated in other studies as a pro-regenerative factor in volumetric muscle loss (VML) after trauma [158,159]. In contrast to this idea, Schiechl et al. showed that basophils promoted the hyperactivation of pro-fibrotic responses in fibroblasts, in a model of chronic cardiac allograft rejection [160]. The effects of IL-4 and other potential therapeutics with varying impacts demonstrate the need for a greater understanding of the implications of therapies for pro- and anti-inflammatory mediators, as several are intertwined in both positive and negative inflammatory responses. 

The impact of biological scaffolds on the immune system is currently believed to be the primary factor responsible for the positive regenerative outcomes [161]. Groups have previously found that biologically derived scaffolds can specifically polarize macrophages to the M2 macrophage state in vitro as well as in vivo [104]. Several mechanisms for this response have been proposed. For example, the breakdown of ECM when used as a scaffold material can reveal multiple cryptic domains of ECM-associated macromolecules, which govern a wide range of cell functionalities involving migration, invasion, adhesion and differentiation [105,162]. The fibronectin sites found naturally in the ECM have also proven critical in regulating cellular repair and wound homeostasis [162]. Several decellularized scaffolds with FDA approval currently exist, including early successes with AlloDermTM [163] and DermACELL™ [164]. In addition, by limiting the xenogenic complications of donor cells, grafted ECM platforms can be re-supplied with healthy anti-inflammatory cellular payloads. Several groups have had success isolating ECM components and transplanting with cells for cutaneous [165] as well as deep tissue [166,167,168] immunomodulatory and regenerative purposes. Some groups have specifically been able to modulate macrophages to an M2 state upon implantation [167], with anti-inflammatory improvements observed from both the ECM scaffold platform and the delivered MSC components. Naturally derived scaffolds have inherent limitations with regard to supply and, at times, cost of fabrication. Synthetic scaffolds may alternatively prove cost effective, but have yet to show a true advantage in terms of mediating inflammation after implantation without further altering the material properties with bioactive coatings.

Several naturally derived materials exist in large supply and are more cost effective than transplanted ECM. Many have intrinsic biocompatibilities as well as biologic cues to support wound healing [169]. Keratin proteins, for example, can be found within a broad variety of animal tissues including: skin, hair, claws, horns, hooves, whale baleen and bird feathers. In terms of biological cues, keratin contains intrinsic amino acid motifs in the form of RGD, LDV and EDS amino acid–binding sites, which are integrin specific and support cellular adhesion [170,171]. Keratin has also been previously exploited for its ability to induce cell differentiation. Previously, human cardiac stem cells (hCSCs) have been shifted to a smooth muscle cell lineage in vitro [172]. Other studies have displayed keratins ability to induce macrophage differentiation, suggesting that it may be exploited as a potential wound healing therapy [106]. Collagen has predominantly been utilized in the form of thin collagen hydrogels or dried into films that are rehydrated prior to use. Some of the earliest FDA approved collagen hydrogels include Apligraf^TM^, one of the earliest bioengineered skin substitutes consisting of a bilayered collagen hydrogel with keratinocytes on one side and fibroblasts on the other [173]. Similar products were conceived with intrinsic antimicrobial properties. Fibrin hydrogels have been used extensively in the last decade in a variety of tissue engineering applications, including engineering of adipose, cardiovascular, ocular, muscle, liver, skin, cartilage and bone tissues. In addition, they have been used for promoting angiogenesis [174]. Clinical applications of hyaluronic acid (HA) and its derivatives include protection of delicate tissues during surgical manipulations. In addition, visco-augmentation involves filling tissue spaces such as skin, sphincter muscles, vocal and pharyngeal tissues with HA to increase tissue volume. This process is often employed in order to prevent adhesion as well as excessive scar formation. While not inherently anti-inflammatory or immunomodulatory, these materials have proven to be sufficient vehicles for immunomodulatory factors while providing beneficial mechanical properties and physical substrates for wound regeneration.

### 5.3. Cell and Cytokine Therapies

Cells and cell-derived factors are considered an effective therapy to treat traumatic inflammation. After an acute skin and muscle injury, numerous cells play dynamic roles interacting within and to other cell types in an extracellular space within the span of 1–4 weeks [175,176]. In addition to cell–cell and cell–matrix interactions, a plethora of pleiotropic factors are secreted in response to an acute injury [65,177,178]. While the immune response is essential for a regulated normal healing, often host cells overcompensates resulting in a surge in inflammatory levels than required. Consequently, there is a delay in healing of the extremity wounds. Immune cell therapy was first indicated to address chronic wounds. Several pre-clinical studies have shown implantation of immune cells by direct application or local injection to promote healing [179,180,181,182].

A recent meta-analysis on the available cell therapies to treat skin wounds showed progenitor cell-based grafts or direct injection of autologous stem cells to stimulate the wound healing process [183]. Following an acute injury, endogenous progenitor cells recruit to wound site and initiate the repair process. In particular, bone marrow derived stem cells (BMSCs) home to the site of injury and influence macrophages, switching them to an anti-inflammatory phenotype, resulting in release of cell surface mediators IL-10 and TGF-β. These mediators augment proliferation of fibroblasts and suppress B and T cell proliferation [108,184]. BMSCs elicits anti-inflammatory properties by preventing the differentiation of monocytes towards antigen presenting immunogenic dendritic cells. BMSCs elicits this function by secreting secretes anti-inflammatory protein TNF-α stimulated gene/protein 6 (TSG-6). The TSG-6 then reduces NF-κB signaling in the resident macrophage. Discerning the immunomodulatory potentials of BMSC’s, they were injected close to the wound and were found to accumulate at the wound site in response to secondary lymphoid tissue chemokines [185]. Another class of stem cells of interest are adipose derived stem cells (ASCs), whose secretome profiles exhibit secretion of a variety of cytokines including an array of pro-inflammatory factors such as IL-6, IL-8, IL-7, IL-11 and TNF-α [186]. However, the major mechanism by which ADSCs modulate inflammation is through secretion of prostaglandin E2 (PGE_2_). PGE_2_ influences macrophages to switch from M1 to M2 phenotype causing them to secrete an anti-inflammatory cytokine IL-10. In addition, PGE_2_ has an inhibitory role over maturation of dendritic cells and a direct effect on the proliferation and cytokine production of T lymphocytes [187,188]. In addition, ASCs are hypothesized to be immune-privileged due to the lack of major histocompatibility complex II (MHC II) cell surface antigen [109]. Due to these reasons, ASCs are considered suitable for clinical application for treatment of a variety of diseases. In the case of associated acute extremity injury involving muscle, the resident muscle progenitor cells, called satellite cells, respond to injury by accumulation of CD3^+^ T cells, and elicit their immunostimulatory effect through the E-type prostanoid receptor 4 (EP4) receptor, similar to ASCs, by secreting PGE_2_ thus augmenting muscle fiber regeneration and strength [189]. It is well known that T cells play an indirect role in angiogenesis via the C-X-C motif Chemokine Receptor 4 (CXCR4), a cognate receptor for stromal cell derived factor 1 (SDF-1), i.e., CXCR12, which is expressed by immature and mature progenitor cells including hematopoietic cells, BMSCs, endothelial progenitor cells (EPCs), ASCs and satellite cells [190,191]. Accumulating evidence show, early expression of CXCR4-SDF1 complex sets the stage for active remodeling by providing pro-angiogenic concoction. In our recent study, ASCs delivered to an acute volumetric muscle loss in rats were found to home within the injured muscle and in the perivascular space on the peri-luminal surface, suggesting early treatment of an acute muscle injury with progenitor cells can modulate wound bed to its pro-regenerative phase [192].

Unequivocally, inflammatory site of an acute traumatic wound is a mileu of a mixture of cells and secreted factors tangled in a nascent matrix. Physiological insights on the dynamics of cytokines and the matrix interplay led to exploration of possible interventions using secreted factors as a possible therapeutic option. In particular, pro-inflammatory and mitogenic growth factors, play a pivotal role in signaling and promoting the healing process [66]. In a rodent study, application of neutralizing antibody to IL-10 inhibited the infiltration of neutrophils and macrophages toward the site of injury, providing evidence for phase-specific control of cellular infiltration in acute wounds [193]. IL-6 and IL-1β are predominant cytokines during early phases of healing, and antibodies targeting these pro-inflammatory cytokines have been shown to positively modulate the inflammatory process. In particular, elevated level of IL-6 following burn injury has been associated with mortality [194]. Modulation of IL-6 to dampen its expression, but not completely stop it, may be important for allowing repair of inflammation following polytraumatic burn injury [195]. Further, we previously showed topical application of antibodies targeting TNF-α or IL-6 could reduce the extension of necrosis by modulating inflammation locally in a partial-thickness rat burn model [196]. IL-1 is another pro-inflammatory cytokine expressed in a wide variety of diseases, ranging from systemic to local conditions. Anti-inflammatory therapies targeting IL-1 in a broad spectrum of diseases has been comprehensively reviewed recently [197]. While several FDA-approved IL-1 inhibitors exist, none specifically targets cutaneous wounds. However, there is lack of evidence for an effective anti-IL-1 treatment option for extremity skin and muscle injury. Another novel approach under investigation to improve treatment of chemical burns is targeting the intracellular serine/threonine kinase substrate downstream from p38 mitogen-activated protein kinase (MAPK). Treatment of cutaneous wounds with MAPK-activated protein kinase-2 inhibitor (MAPKAPK2i or MK2i) has shown to markedly decrease the mRNA levels of a chemokine, macrophage inflammatory protein-1 Alpha (MIP-1α) and pro-inflammatory cytokines IL-6 and IL-1β [198,199].

Clinically, topical treatments with macrophages [200], granulocyte-macrophage colony-stimulating factor (rhGM-CSF) [201] and endothelial progenitor cells (EPCs) [202] have shown success in diabetic wounds. However, there are very few clinical studies that have been conducted using application of immune cells or cell-derived factors to treat acute skin wounds. Of note, topical application of rhGM-CSF hydrogel on deep second burn wounds was successfully evaluated and improvement in healing was observed for 4 weeks [203]. In another similar study, rhGM-CSF application showed higher healing rates than those in the placebo group (*p* < 0.01). The wound healing time in the rhGM-CSF group (18.8 ± 7.6 days) was significantly shorter than that in the placebo group (25.5 ± 4.6 days, *p* < 0.01) [204]. In addition, clinical studies has shown treatment of burn wounds with rhGM-SF has significantly affected the scores of periwound inflammation, wound purulence and discharge [205]. 

### 5.4. Cell Secretome and Extracellular Vesicles

The application of cellular therapies though exciting has posed hurdles in the FDA approval-path to biologics. Though the cellular approach towards immune modulation has proven beneficial in vivo, still, there is a demand to identify alternatives to the cellular therapies. Of note, recent approaches have also attempted to remove the cell from cellular therapies, focusing on secreted cell factors to modulate acute wound inflammation. This process has the advantage of removing costly and/or time-consuming cell culture, limiting xenogenic transplant complications and circumventing the need to introduce even small populations of apoptotic cells which inevitably occurs during transplantation and may further exacerbate the pro-inflammatory response. Deeper understanding of the complex cellular interactions with the host, combined with the identification of more molecular targets and secretomes from cells, has opened new avenues on how they benefit wound healing [206]. Extracellular vesicles (EV), also known as secretomes or exosomes, are self-contained vesicles characterized by the absence of a nucleus which are released by cells into the extracellular space. EVs are characterized by their specific payloads which may be composed of DNA, mRNA, microRNAs or a milieu of biologically active proteins [207,208]. This cargo is protected within a lipid bilayer, allowing for advantageous methods of storage and transport. 

EVs contribute to nucleic acid-based immunomodulation due to their payloads consisting of DNA and RNA that is complementary to wound healing. Nakamura et al. showed the presence of myogenic miRNAs miR-1, miR-133, miR-206 and miR-494 in MSC-EVs as well as the conditioned media of the same cells [209]. Each of these miRNAs has been shown to induce a protective effect against ischemia-induced muscle trauma [122,210]. Interestingly, it was reported that miRNA encapsulated in EVs appeared to have enhanced functions when compared with miRNA released into the conditioned media. This observation has been noted elsewhere as well [211]. 

To date, clinical studies employing EVs are limited and scarcer still with regard to application as a therapy for traumatic injuries; however, in vitro and in vivo studies have produced promising results thus far. In a mouse model of cardiotoxin-induced (CTX) muscle injury and wound homeostasis mediated by EVs from human amniotic fluid derived mesenchymal stromal cells (AF-MSCs), the anti-inflammatory activity, ability to enhance cellular proliferation, and the capacity to protect against cellular senescence were all found to be increased in EV treated injuries. Mechanistically, these improvements were found to be mediated, at least in part, through the repression of the NF-κB pathway [111]. In another mouse model of CTX-induced muscle injury and angiogenic repair, matrigel plugs containing EVs secreted from adipose tissue derived mesenchymal stromal cells (AT-MSCs) were observed 3 weeks after injury and material implantation. Significantly increased vasculature was observed at the periphery of the plug in EV treated mice. To support the clinical use of EVs after severe trauma, LoSicco et al. observed under hypoxic conditions EVs isolated from MSCs were able to upregulate the expression of several miRNA implicated in muscle repair, in particular miR-223, miR-146b, miR-126 and miR-199a. The effects were attributed to increased pro-angiogenic factors platelet and endothelial cell adhesion molecule-1 (PECAM-1) and vascular endothelial growth factor-A (VEGFA) [110]. 

## 6. Immune Engineering Approaches to Modulate Inflammation

Immunoengineering represents the discipline of bioscience and technology that enhances antigen presentation, revive innate immunity, delivery of active therapeutic to specifically modulate immune cells, ranging from synthetic drug to biologics-including cells and cellular factors, and implementing engineered biocompatible materials to unravel the immune system function and regulation in health and disease [212,213]. Recent advance in immune biology, analytical and engineering tools has allowed deconvolution of complex immune function discerning to a variety of diseases, resulting in the development of novel therapies to specifically modulate and control dysregulated immune functions and restore normal physiological state. Several therapeutic options have been researched, including, cellular engineering, cytokine therapy, nucleic acid-based immunotherapy, synthetic drug delivery, and bio-engineered material based approaches (Figure 2). The following sections shed light on the recent advancements on the current and emerging immune engineering approaches developed to target acute immune responses following a traumatic insult to the skin and the underlying muscle. Additionally, a summary of select immune engineering approaches and their applications can be found in Table 3. 

### 6.1. Nucleic Acid and Aptamers Based Immune Targeting

The advent of DNA nanotechnology has paved the way for exploration of its biological application, including tissue regeneration, cancer therapy, inflammatory diseases, imaging, diagnosis, drug delivery and therapeutics [218]. Due to non-immunogenic deoxyribonucleic basic component, DNA nanoparticles present low immunogenicity and their internalization would not intensify immunoreaction. DNA nano particle have been widely applied in tissue regeneration and immune stimulation. DNA-encoding VEGF were designed to target inflammation in both chronic and acute wounds. A gene-activated bilayer dermal equivalents (Ga-BDEs) developed by loading the nano-sized complexes of Lipofectamine 2000/plasmid DNA-encoding VEGF into a collagen-chitosan scaffold/silicone membrane bilayer dermal equivalent was shown to have a dual functions of immunomodulation and pro-angiogenesis simultaneously [219]. Magnetic DNA nanospheres containing expression plasmids DNA (pDNA) encoding VEGF was able to promote angiogenesis in ischemic limb by alleviating the high oxidative stress and inflammatory micro-environment in both mouse and rabbit models [214,215]. In another study, pDNA-VEGF accelerated excisional burn wound healing, by inhibiting inflammation response; IL-1β or TNFα expression were significantly reduced, thus promoting microvascular formation [216]. The advantage of DNA nanoparticle is it can be conveniently designed to desired shape, to include single layer, wired frame and multilayer structures. This versatility has led to the design of advanced DNA nano materials such as multifunctional and intelligent DNA nano device, nano flowers (NFs), nano circuits and nano robots capable of targeting and delivering payloads such as drugs, fluorescent optical labels and even aptamers [220,221,222,223,224]. 

Single stranded DNA (ssDNA) or aptamers, are a special class of nucleic acid molecules can form secondary and tertiary structures capable of specifically binding proteins or other cellular targets [225,226]. Aptamers are selected by a process called systematic evolution of ligands by exponential enrichment (SELEX), in which DNA or RNA molecules are selected by their ability to bind their targets with high affinity and specificity, comparable to those of antibodies [227,228]. Harnessing the selective binding ability with specific target at high precision, aptamers are designed to interact with complementary molecules targeting the immune system. Following an acute injury, exposure of wound surface to any bacterial pathogens activates antigen-specific acquired immunity signaling pathways such as MAPK and nuclear factor-kB (NF-kB) via the activation of Toll-like receptors (TLRs) on neutrophils, monocytes, macrophages as well as B cells and T cells [229] favoring the release of inflammatory cytokines. There are aptamers specifically that bind to TLRs (2, 9) and cytokines such as IL-6 receptor (IL-6R), IL-10R to dampen the severity of cytokine storms [230,231]. Thus, far most of the aptamers are designed to address pathological conditions such as cancer, atherosclerosis, immunodeficiency and autoimmunity [232,233,234,235]. In the arena of wounds and traumatic injuries, aptamers are used as a potential molecular probes to examine bacterial infection, thus allowing the remote detection of a pathogens [236,237]. Recently, a much more advanced point-of-care in situ platform is developed to monitor wound status beyond detection of pathogenic bacteria. This device comprises flexible multiplexed immunosensors integrated with aptamers sensor array for measuring inflammatory mediators such a TNFα, IL-6, IL-8 and TGFβ1, and *Staphylococcus aureus*. Additionally, the array can monitor vital parameters at the wound site such as temperature and pH. The entire immune-platform is a microfluidic device capable of monitoring each of the aforementioned analyte through wound exudate sample collector. More importantly the entire platform is built on flexible electronics for wireless, smartphone-based data readouts [238].

### 6.2. Theranostics Immune Targeting

A recent symposium conducted by the National Academy of Engineering on leading-edge engineering technology identified immune theranostics as one the promising and innovative technological advancements to harness the full potential of immunotherapy in the treatment of a wide range of inflammatory disorders [239]. Theranostics is an engineering approach that combines delivery of therapeutic and diagnostic agents. Theranostic immunotherapy focuses on development of nanoscale biomaterials (NBMs) to modulate the immune system [240,241]. Conceptually, theranostic NBMs are custom targeting nano-sized materials tethered with antibodies, peptides, aptamers and other molecular recognition motifs [242]. The current theranostic NBMs are primarily used to target inflammation-driven pathologies. These immune NBMs specifically directs the immune balance toward either a pro- or anti-inflammatory state depending on the desired outcome for a given disease [243]. Theranostic hydrogel approach to improve acute wounds has been recently attempted. A sophisticated hydrogel from chemically modified hyaluronic acid (HA), dextran (Dex) and β-cyclodextrin (β-CD) was designed to deliver VEGF plasmid as the anti-inflammatory and pro-angiogenic components. The hydrogel accelerated the splinted excisional burn wound healing by inhibiting inflammation response and the pDNA-VEGF promoted new patent blood vessel formation characterized by co-localized positively stained CD31 and α-SMA cells within the wound bed [216]. While the theranostic approach to treat acute injury is still in infancy, still underlying mechanistic of an NBM-ligand-mediated inflammatory targets could be applied to design novel immune-theranostics to regulate inflammatory status of an acute injury.

The rising need of personalized medicine to deliver on-demand drugs has led to development of strategies to engineer materials that will respond to local changes in the wound and release therapeutics. In a recent study, a smart flexible electronics-integrated hybrid wound dressing was developed integrating a polydimethylsiloxane-encapsulated flexible electronics with a temperature sensor and ultraviolet (UV) light-emitting diodes, and polyethylene glycol diacrylate (PEGDA) hydrogel loaded with gentamycin responsive to UV activation to treated infected 3 cm diameter full thickness model. The developed smart dressing was capable of monitoring temperature and using a NIR sensor detects infection induced hyperthermia. Subsequently, the integrated LED triggers antibiotic release upon UV activation [217]. In another recent research study, a smart wound dressing features glowing nano sensors with fluorescent magnesium hydroxide nano sheets (Mg(OH)_2_-NS) was developed. Magnesium’s antimicrobial, anti-inflammatory and biocompatible properties are well known. The developed smart dressing with capability to mitigate medically relevant bacterial and fungal infections were doped with pH probe to monitor wound status in real time [244]. 

The next generation of theranostics will include a data-driven wound healing assessment and management system by leveraging machine-learning and deep-learning frameworks. Integrating immune engineering, smart materials, bioelectric technologies will enable monitoring of temperature, moisture, pressure, pH and cytokine, and will also enable precise, on-demand release of therapeutic for better wound management and healing. 

## 7. FDA Position Statement

For successful clinical translation, one should adopt standard operating procedures designed to generate immunotherapeutics under current good manufacturing practice regulations. The FDA’s Center for Devices and Radiological Health (CDRH) classified wound dressings combined with drugs as “wound dressings containing drugs”, under product code “FRO.” These products include solid wound dressings, gels, creams, ointments and liquid wound washes. Within this classification, wound dressings combined with a drug are generally regulated as combination products. By definition, a combination product is comprised of two or more constituent parts (i.e., drug/device, biologic/device, drug/biologic or drug/device/biologic) and should meet the requirements of a combination product under FDA 21 Code of Federal Regulation (CFR) 3.2. There are several COX-2 selective NSAIDs, and prescription or over-the-counter (OTC) non-selective NSAIDs for use to treat a variety of general ailments. Some of the examples of COX-2 selective agents include Celecoxib, Valdecoxib and Rofecoxib. However, after approval, the FDA made strongly worded black-box warnings for each of the three COX-2 inhibitors currently approved in the United States [245]. A quest to identify specific approved anti-inflammatory drug and drug eluting dressing followed, and in 2016, under 21 CFR 3.2, the FDA recommended ingredients that are contained within unclassified and cleared wound dressings. Within the comprehensive list of drugs, both inorganic and organic chemical constituents are included. The wound dressing Fortaderm^®^, for example, is a collagen-based polyhexamethylene biguanide (PHMB) antimicrobial wound dressing that gained FDA approval in 2001. Other chemical drugs listed are magnesium-based inorganic compounds, for example, magnesium oxide and sulfate. Owing to the antimicrobial efficiency of magnesium, magnesium hydroxide-based compounds and magnesium doped dressings are considered for smart dressings with the capability to mitigate medically relevant bacterial and fungal infections [244].

The FDA has a Center for Biological Evaluation and Research (CBER), which regulates human cells, tissues and cellular and tissue-based products (HCT/P). FDA code and the definition of the use of cells as therapeutics under biologics are complex. Our recent review provides a succinct mechanism of FDA approval mechanism of drugs, biologics and medical devices for wound healing purposes [246]. Wherein, drugs and biologics (i.e., stem cell therapies) take the longest time to receive an FDA approval, requiring both pre-clinical animal studies followed by three phases of clinical trials. While there are a number of clinical trials initiated to treat acute wounds with the primary aim of skin regeneration and closure, curb-siding inflammation has been a secondary point of determination [246]. Still, there is a long way to go for an exclusive cell therapy specifically addressing modulation of the inflammatory response of an acute injury. To date, stem cell-based therapies are the most likely candidates to show promising result in clinical trials.

The FDA position also stands true for cell secretomes, due to hurdles in clinical translation of the complex EV populations. A true success of EV research relies on manufacturing and successful application to treat human etiologies, while being in compliance with existing regulatory frameworks. Strategies for a methodical, safe and efficacious EV scale-up manufacturing and pharmaceutical use have been laid out as positional statements by the members of the International Society for Extracellular Vesicles (ISEV) and of the European Cooperation in Science and Technology (COST) program of the European Union. As the classification defines “subsequent requirements for manufacturing, quality control and clinical investigation”, it is of major importance to define if EVs are considered the active drug components or they primarily serve as drug delivery vehicles. Taking this into consideration, this important FDA guideline requires that a product should be manufactured under the current good tissue practice (CGTP) by establishments that perform a manufacturing step under contract, agreement or other arrangement for another HCT/P establishment. The core CGTP requirements include: facilities, environmental control, equipment, supplies and reagents, recovery, processing and process controls, storage, receipts, pre-distribution shipment and distribution of HCT/P, and donor eligibility determinations, screening and testing. To qualify for clinical usage, EVs typically follow a sequential step, which are, EV-based investigation medical product (IMP)/Investigation new drug (IND) filing, potency evaluation in vitro and in vivo according to the CFR of the FDA, route of administration (systemic versus local application), single versus multiple administration and dosage of the IMP/IND per treatment, identification of personalized versus common (off-the-shelf) use, source cells and their current good manufacturing practice (GMP) production documentation, naturally or endogenously loaded versus artificially or externally loaded EVs. Normally, EV purification is performed using tangential flow filtration (TFF) combined with a short ultracentrifugation (UC) step [247,248]. A thorough quality control (QC) and documentation of the final product testing, including characterization data, and batch-to batch consistency records are pre-requisites to achieve the approval for clinical testing [249,250]. In addition, the GMP manufacturing method assures high exosome yield (>10^13^ particles) and consistent removal (≥97%) of contaminating proteins [251]. To date, EVs are under clinical investigation, and a comprehensive list of on-going trials are published recently in the position statement article published by EVOLVE France, (Extracellular Vesicle translatiOn to clinicaL perspectiVEs), created in 2020 [252,253]. 

In summary, cells and EVs are categorized as “biological medicinal products” and if cells and EVs are delivered through a biomaterial, such a product will then be classified as a Class III medical device. With committed regulatory guidelines, the future development of the cell/EV-based medicinal products show promises to come closer to patients while maintaining quality, safety and efficacy. 

## 8. Conclusion and Future Directions

Physiological immune function is a complex phenomenon involving various elements from a variety of cellular and nuclear factors. An external insult, such as traumatic acute injury, causes changes to the finely balanced network of events leading to an alternation of the normal immune function. The primary host-response is to resolve and restore this imbalance to a normal physiological state. Due to the severity and acute exacerbation of inflammatory reaction, there is a race against time to regulate this sudden surge. In particular, acute injury inflammation spans a short time, and if not restored may lead to an aberrant healing response. It is well-recognized that inflammation is essential for host defense, but prolongation or perturbation can be deleterious. The field of immune-modulation has evolved over years to address various etiologies involving immune dysfunction. This article provides a discussion of therapeutic approaches available to specifically address acute soft tissue injury. The current therapeutic options—drugs, biologics and wound dressings—modulate the inflammation by acting upon a group of inflammatory mediators and immune cell infiltrate to the injury site. With greater understanding of acute phase inflammatory network, and the variety of pleiotropic factors involved, approach towards treating an acute injury inflammation has shifted focus towards a more targeted approach. With the advent of burgeoning immune engineering field several technologies are in the horizon with a prospective to address the needs of precision medicine. Hybrid biomaterials are a promising next step in regenerative medicine and will likely be able to combine the benefits of different materials embedded with specific immune-targeting agents/molecules. While the regulatory processes for these newer technologies are complex and perhaps not completely defined by the FDA, there is still huge promise to revolutionize the treatment strategies to treat acute and complex extremity injuries by tuning a fine balance to the inflammatory phase to proceed towards a near normal healing process.

## Figures and Tables

**Table 1:** A compilation of the essential growth factors, their cell source and functions during the wound healing process.

Growth Factors	Cell Source	Functions	Ref.
Fibroblast growth factor 2 (FGF2)	Keratinocytes Mast cells Fibroblasts Endothelial cell Smooth muscle cells Chondrocytes Macrophages T cells	Chemotactic for fibroblasts; Mitogenic for fibroblasts and keratinocytes; Stimulates keratinocyte migration, angiogenesis wound contraction and matrix production.	[65,66]
Epidermal growth factor (EGF)	Platelets Activated macrophages Fibroblasts	Mitogenic for keratinocytes and fibroblasts; Stimulates re-epithelialization and granulation tissue formation.	[65]
Platelet derived growth factor (PDGF)	Platelets Keratinocytes Macrophages Endothelial cells Fibroblasts	Chemotactic for neutrophils macrophages, fibroblasts and smooth muscle cells; Stimulates production of matrix metalloproteinases, fibronectin and hyaluronic acid; Stimulates angiogenesis.	[65,67,68,69,70]
Transforming growth factor β (TGFβ)	Platelets Keratinocytes Macrophages Lymphocytes Fibroblasts	Most important factor in wound healing; Maintains monocyte chemotaxis, fibroblast migration and differentiation; Angiogenesis and fibronectin synthesis; Regulates increased synthesis of collagen and extracellular matrix and decreased degradation by matrix metalloproteinase.	[65,71,72,73,74]
Vascular Endothelial growth factor (VEGF)	Platelets Neutrophils Macrophages Endothelial cells Smooth muscle cells Fibroblasts Mesenchymal cells	Increases vascular permeability; Mitogenic for endothelial cells.	[65,75,76]

**Table 2:** Summary of selected immunomodulatory approaches to promote wound healing.

Approach	Injury Type	Outcomes	Limitations	Ref.
***Pharmacological agents***				
NSAIDs	Debrided combat-related extremity wounds	Nonsteroidal anti-inflammatory drug treated group had significantly decreased concentrations of inflammatory cytokines, interleukin-2 (IL-2), interleukin-6 (IL-6), interleukin-8 (IL-8) and monocyte chemoattractant protein-1 (MCP-1).	Patients represented a very specific cohort of injuries (blast injury). Frequency of debridement operations may have skewed some results.	[97]
COX-2 Inhibitor (Celecoxib)	Sciatic Nerve Crush	In comparison with control group, celecoxib treatment had significant beneficial effects on sciatic functional index (SFI), with a significantly better score on day 7.	Small sample size and large data variability.	[98]
Skeletal muscle ischemia/reperfusion (I/R) injury	Inducible nitric oxide synthase (iNOS) inhibitor (1400W)	1400 W markedly improved the recovery speed of vessel diameter and blood flow.		[99]
Manganese superoxide dismutase (MnSOD) mimetic molecule, MnE	Dermal full-thickness excision injury	MnE significantly advanced wound closure by two days. MnE regulated antioxidant defense systems.		[100]
Injectable curcumin-loaded Zn-Al layer double hydroxide nanocomposites	Intramuscular implantation	Curcumin and Curcumin Nano hybrid revealed good tissue repair in acute and chronic wounds with good bio-compatibility and healing activity with collagen formation.		[101]
***Biological and synthetic platforms***				
Modification to surface topography and hydrophilicity	In vitro, neutrophil activation and macrophage polarization	Hierarchy of least-to-greatest pro-inflammatory cytokine secretion: Rough Hydrophilic surfaces → Rough surfaces → Smooth surfaces Hierarchy of least-to-greatest anti-inflammatory cytokine secretion: Smooth surfaces → Rough surfaces → Rough Hydrophilic surfaces Hierarchy of least- to-greatest inflammatory cell secreted factors (CXCL-10, MCP-1): Rough Hydrophilic surfaces → Rough surfaces → Smooth surfaces.	In vitro work may have provided a limited example to determine actual mechanisms of action compared to in vivo studies.	[102]
Heparin-immobilized copolymers of L--lactide (LA) and 5-methyl-5-benzyloxycar-bonate-1,3-dioxan-2-one (MBC) on metal stents	Porcine coronary artery injury model	Heparinized copolymers effectively reduced platelet adhesion and protein adsorption while increasing the plasma recalcification time and thromboplastin time in vitro.	No in-stent thrombosis was observed in any stenting groups. The efficacy of heparinized copolymers in reducing the rate of thrombosis was not tested.	[103]
Biologically derived surgical mesh materials	In situ polarization of macrophages responding to implanted mesh materials	There was a strong correlation between the early macrophage response to implanted materials and the outcome of tissue remodeling. Increased numbers of M2 macrophages and higher ratios of M2:M1 macrophages within the site of remodeling at 14 days were associated with more positive remodeling outcomes.	Limited and potentially non-specific surface markers for macrophage characterization were employed. No efforts to quantify M1 and M2 macrophages concurrently.	[104]
Dermal ECM (D-ECM) or Urinary bladder matrix ECM (UBM-ECM) coating polypropylene mesh	In vivo macrophage polarization following mesh implantation in a rodent model	Uncoated polypropylene mesh elicited a greater M1 response at the mesh fiber surface, which was decreased by each ECM coating type beginning at 7 days. Diminished M1 response was accompanied by a reduction in the number of foreign body giant cells at 14 and 35 days.	M1 and M2 macrophages were identified by single surface markers, markers of other macrophage subtypes were not considered.	[105]
Keratin and Collagen coatings (films)	In vitro macrophage polarization	Exposure of macrophage cell line to keratin biomaterial substrates prompted a shift toward M2 phenotype Collagen control surfaces produced both M1 and M2 macrophage populations.		[106]
***Cell and cytokine therapies***				
Macrophage polarization	In vitro model—monocytes embedded in modified hydrogel	Increased number of M2 macrophages.	M2 macrophages released large amounts of pro-inflammatory cytokines.	[107]
Mesenchymal stem cells (MSCs)	Mouse lethal radiation injury	MSC influenced macrophages showed a distinct gene expression profile that positively correlated with pathways that promote tissue repair. MSC influenced macrophages enhance survival of mice experiencing radiation injuries.		[108]
Human bone maow stromal cells (BM-SC)	Specialized in vitro culture for modulating cell phenotype	Adipose tissue-derived stromal cell protein expression phenotype was similar to that of human bone marrow stromal cells. Cells cultured under adipogenic or osteogenic conditions promoted differential expression of growth stimuli.		[109]
***Cell secretome and extracellular vesicles***				
Mesenchymal Stem Cells (MSC) Extracellular vesicles (EVs)	Bone marrow-derived macrophage polarization, Cardiotoxin-induced skeletal muscle injury	MSC EVs elicited a significant switch from a M1 to a M2 macrophage phenotype. MSC EVs in vivo contributed to decreased IL-6 and NOS2 with increased myogenic markers (Pax7, MyoD and Myhc).		[110]
Amniotic fluid stem cell-derived extracellular vesicle	Cardiotoxin induced tibialis anterior mouse muscle injury	Secretomes were capable of promoting cell proliferation, migration and protection from senescence in vitro. Secretomes promoted muscle regeneration in vivo.		[111]

**Table 3:** Summary of selected immune engineering approaches to promote wound healing.

Approach	Injury Type	Outcomes	Ref.
***Nucleic acid and aptamers based immune targeting***			
Intra-arterial VEGF gene delivery by magnetic DNA nano spheres	Rabbit limb ischemia model	VEGF delivery promoted angiogenesis and arteriogenesis in ischemic limbs by alleviating the high oxidative stress and inflammatory micro-environment.	[214]
Nanoparticle-based pcDNA3.1-CYP2J2 plasmid DNA (pDNA) delivery system (nanoparticle/pDNA complex)	Mouse limb ischemia model	Improved inflammatory micro-environment; angiogenesis and muscle repair.	[215]
Hydrogel loading plasmid DNA encoding VEGF	Mouse burn wound model	pDNA-VEGF accelerated excisional burn wound healing by inhibiting inflammatory response. Specifically, IL-1 β or TNF- α expression were significantly reduced, thus promoting microvascular formation.	[216]
***Theranostics immune targeting***			
Smart flexible electronics-integrated wound dressing	Pig full thickness wound model	Wound status in real time was monitored. Bacterial infection was detected and wounds were effectively treated	[217]

## Data Availability

Not applicable.
